# Escape from senescence: revisiting cancer therapeutic strategies

**DOI:** 10.1080/23723556.2022.2030158

**Published:** 2022-02-15

**Authors:** Christos P. Zampetidis, Argyris Papantonis, Vassilis G. Gorgoulis

**Affiliations:** a Molecular Carcinogenesis Group, Department of Histology and Embryology, Faculty of Medicine, National Kapodistrian University of Athens, Athens, Greece; b Biomedical Research Foundation, Academy of Athens, Athens, Greece; c Translational Epigenetics Group, Institute of Pathology, University Medical Center Göttingen, Göttingen, Germany; d Center for Molecular Medicine Cologne (CMMC), University of Cologne, Cologne, Germany; e Division of Cancer Sciences, School of Medical Sciences, Faculty of Biology, Medicine and Health, University of Manchester, Manchester, UK; f Center for New Biotechnologies and Precision Medicine, Faculty of Medicine, National and Kapodistrian University of Athens, Athens, Greece; g Faculty of Health and Medical Sciences, University of Surrey, Surrey, UK

## Abstract

Although senescence has been considered as an irreversible cell arrest state, accumulating evidence challenge this view. Consequently, senescence appears as an imperfect barrier to impede cancer progression, constituting a step prior to disease relapse. Therefore, cancer treatment strategies may benefit if revisited to include senolytic agents.

Cellular senescence (thereafter senescence) is a cell state triggered in response to various stressors, including among others, telomere attrition, oncogene activation, oxidative stress and exposure to genotoxic agents.^1^ Senescence is mainly characterized by an irreversible cell cycle arrest and a specific secretory phenotype, known as senescence-associated secretory phenotype (SASP).^1^ Interestingly, although senescent cells are considered to have lost their proliferation capacity, they still remain metabolically active and communicate with their microenvironment either indirectly through SASP or directly by forming cytoplasmic bridges.^1^ Activated oncogenes can elicit a distinct type of senescence, known as oncogene-induced senescence (OIS), which constitutes a tumor-suppressive reaction elucidated in our previously proposed “Oncogene-induced DNA damage model for cancer development”.^2^ In this way, the organism attempts to counteract the malignant transformation of normal cells.

Nevertheless, OIS plays an ambiguous role in cancer development. Accumulating data concur that OIS is a double-edged sword as it can also promote cancer progression under certain circumstances.^1^ This can be mediated by pro-tumorigenic factors secreted from senescent cells in the context of SASP. However, although senescence has been considered as an irreversible cell arrest state, our group recently challenged this perception and set the basis for the escape from oncogene-induced senescence concept (EOIS).^1,3^ To functionally study this phenomenon, we developed cellular models in which deregulated oncogenic and onco-suppressor networks resulted in EOIS, appearance of proliferating cells with aggressive traits and elevated genotoxic drug tolerance.^1,3^ Considering these findings, an emerging question was how EOIS could be explained in the context of our model,^3^ as the mechanistic aspect remained unaddressed.^1,3^

For this purpose, we exploited our recently generated Cell Division Cycle 6 (CDC6)-overexpressing normal cellular epithelial system, as the CDC6 replication licensing factor possesses potent oncogenic properties^4^ (and references therein). Notably, CDC6 mediated genomic instability, triggered genetic alterations, which were followed by chromatin reshaping and remodeling during EOIS.^4^ Among the accumulated genetic rearrangements, we observed a recurrent inversion on chromosome 3. This event, in combination with the consequent reshuffling of the regional chromatin, resulted in reactivation of a dormant replication origin and upregulation of Basic Helix-Loop-Helix Family Member E40 (*BHLHE40*), a transcription factor that is involved in the regulation of circadian clock machinery.^4^ Given that increasing amount of data connectthe circadian clock with the coordination of the cell cycle, deregulations of circadian rhythm effectors might in turn disrupt the physiological cell cycle process conferring a detrimental impact at the organism level.^5^ The latter is reflected in various pathological processes, setting the grounds for further investigation. Considering also that the link between replication and transcription is an ancestral process,^6^ an additional emerging question is how chromatin rearrangements can reactivate origins of replication affecting gene transcription. Hence, defining the molecular network that associates the circadian rhythm, cell cycle regulation and gene expression and its impact on patho-physiological processes, is a promising future endeavor that deserves to be pursued. Interestingly, the International Agency for Research on Cancer (IACR) recently classified night shift work as “probably carcinogenic to humans”,^7^ as the nature of this work might deregulate the physiology of the circadian clock machinery.

Beyond these mechanistic aspects, cancer treatment protocols have so far been developed with the scope of targeting proliferating cells. Although current chemotherapeutics eliminate a subset of cancer cells, remaining cells can enter therapy-induced senescence^8^(Figure 1). However, such treatments are not efficient against non-proliferating senescent cells and hence those cells constitute a source of recurrence. Unfortunately, *in vitro* data suggest that following treatment with chemotherapeutic compounds, a subset of senescent cells evades therapy-induced senescence^1,8^(Figure 1). Interestingly, the emerging clones are resistant to the agent that originally triggered senescence, representing the *in vivo* analogue of cancer relapse after an initial response to treatment (Figure 1). In support of the above, in Hodgkin lymphoma patients exhibiting a high frequency of senescent cells we showed a correlation with adverse outcome conferred by resistance to therapy.^9^

**Figure 1.**
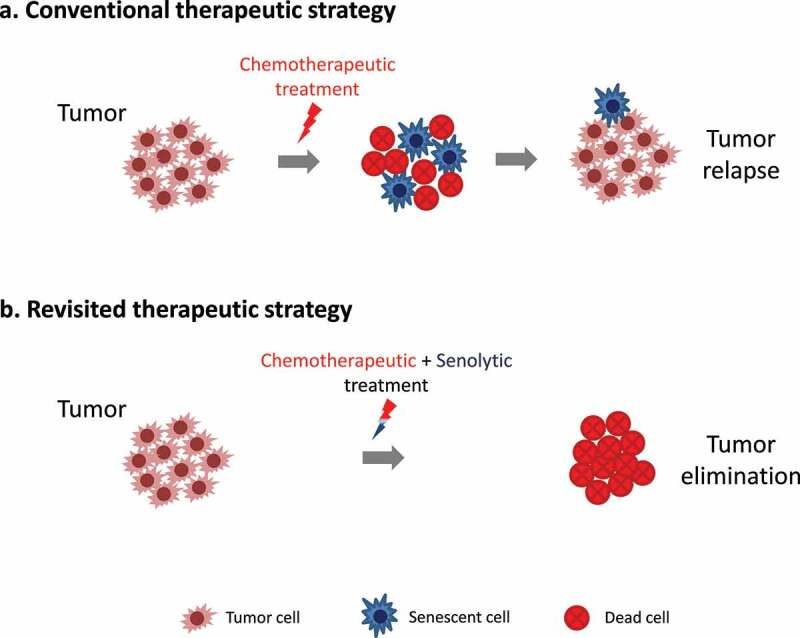
Revisiting cancer treatment modalities. (a) Conventional therapeutic strategies can eliminate a subpopulation of proliferating cancer cell, but induce senescence in other cells allowing them to evade eradication. Subsequently, these cells can lead to tumor relapse, upon escape from senescence. (b) Revisiting conventional therapeutic strategies to include also senolytic agents may be beneficial, since cells undergoing therapy-induced senescence are also eradicated, thus preventing tumor relapse.

**Alt-text**: A schematic presentation showing that chemotherapeutic treatment triggers tumor cell death and senescence induction. If these therapy-induced senescent cells are left untreated, then they constitute a source of recurrence. Thus, combinatorial treatment with senolytic drugs eliminates also the remaining senescent cells, resulting in complete tumor eradication.

Overall, senescence seems to be an imperfect barrier to impede cancer progression, constituting a step prior to disease relapse. Thus, eliminating senescent cells can be of major clinical importance, as they can potentially be a source of regression (Figure 1). Lately, there is an increasing interest in agents capable of removing senescent cells from the organism, known as senolytic drugs. Those drugs induce apoptosis selectively in senescent cells. Therefore, implementation of senotherapeutic agents against neoplasms, like in the case of Hodgkin lymphomas,^9^ where escape from senescence may actively contribute to cancer progression is a treatment avenue that needs to be deeply investigated. Additionally, little is known about mechanisms that confer apoptosis resistance to senescent cells. Elucidating factors, which are responsible for cell death resistance, will unlock additional possibilities in the field of senolysis and provide more powerful means to fight cancer.

Last but not least, senolysis may also have implications in diseases other than cancer. Particularly, SARS-CoV-2 induces senescence in infected cells.^10^ These cells express SASP factors, but also exhibit increased expression of the Apolipoprotein B mRNA Editing Catalytic Polypeptide-like (APOBEC) RNA-editing enzymes as a defense reaction.^10^ As senescent cells have a prolonged lifespan, this contributes to an increased mutational burden in the viral genome, thereby facilitating the generation of new viral strains.^10^ Thus, the infected senescent cells constitute the “factory” in which new viral strains may be produced. However, other classes of viruses are not capable of inducing senescence. This is a paradoxically beneficial effect, as the lower mutational burden contributes to the genomic stability of these viruses, which in turn facilitates the development of effective vaccines. On the other hand, the treatment of senescence-inducing viruses might benefit from the application of senolytic drugs as a means to impede the emergence of new viral variants and thus increase the effectiveness of the vaccination programs.

## References

[1] Gorgoulis V, Adams PD, Alimonti A, Bennett DC, Bischof O, Bishop C, Campisi J, Collado M, Evangelou K, Ferbeyre G et al. Cellular Senescence: Defining a Path Forward. Cell. 2019;179(4):813–3. PMID:31675495. 10.1016/j.cell.2019.10.005. 31675495

[2] Halazonetis TD, Gorgoulis VG, Bartek J. An oncogene-induced DNA damage model for cancer development. Science. 2008;319(5868):1352–1355. PMID:18323444. 10.1126/science.1140735. 18323444

[3] Komseli ES, Pateras IS, Krejsgaard T, Stawiski K, Rizou SV, Polyzos A, Roumelioti FM, Chiourea M, Mourkioti I, Paparouna E et al. A prototypical non-malignant epithelial model to study genome dynamics and concurrently monitor micro-RNAs and proteins in situ during oncogene-induced senescence. BMC Genom. 2018;19(1):37. PMID:29321003. 10.1186/s12864-017-4375-1. PMC5763532 29321003

[4] Zampetidis CP, Galanos P, Angelopoulou A, Zhu Y, Polyzou A, Karamitros K, Kotsinas A, Lagopati N, Mourkioti I, Mirzazadeh R et al. A recurrent chromosomal inversion suffices for driving escape from oncogene-induced senescence via subTAD reorganization. Mol Cell. 2021;81(23):4907–4923.e8. PMID:34793711. 10.1016/j.molcel.2021.10.017. 34793711

[5] Gaucher J, Montellier E, Sassone-Corsi P. Molecular cogs: interplay between circadian clock and cell cycle. Trends Cell Biol. 2018;28(5):368–379. PMID:29471986. 10.1016/j.tcb.2018.01.006. 29471986

[6] Fisher D, Mechali M. Vertebrate HoxB gene expression requires DNA replication. Embo J. 2003;22(14):3737–3748. PMID:12853488. 10.1093/emboj/cdg352. 12853488 PMC165622

[7] Ward EM, Germolec D, Kogevinas M, McCormick D, Vermeulen R, Anisimov VN, Aronson KJ, Bhatti P, Cocco P, Costa G et al. IACR Monographs Vol 124 group. Carcinogenicity of night shift work. Lancet Oncol. 2019;20(8):1058–1059. PMID:31281097. 10.1016/S1470-2045(19)30455-3. 31281097

[8] Saleh T, Tyutyunyk-Massey L, Gewirtz DA. Tumor cell escape from therapy-induced senescence as a model of disease recurrence after dormancy. Cancer Res. 2019 ;79(6):1044–1046. PMID:30803994. 10.1158/0008-5472.CAN-18-3437. 30803994

[9] Myrianthopoulos V, Evangelou K, Vasileiou PVS, Cooks T, Vassilakopoulos TP, Pangalis GA, Kouloukoussa M, Kittas C, Georgakilas AG, Gorgoulis VG. Senescence and senotherapeutics: a new field in cancer therapy. Pharmacol Ther. 2019;193:31–49. PMID:30121319. 10.1016/j.pharmthera.2018.08.006. 30121319

[10] Karakasiliotis I, Lagopati N, Evangelou K, Gorgoulis VG. Cellular senescence as a source of SARS-CoV-2 quasispecies. Febs J. 2021. PMID:34653312. 10.1111/febs.16230. 34653312

